# Genome sequence of cluster B1 *Mycobacterium smegmatis* phages Thunderbird, Gwilliam, and Jiraiya

**DOI:** 10.1128/mra.00393-25

**Published:** 2025-06-30

**Authors:** Atalie B. Bogh, Jacob D. Gwilliam, Peter Mourik, Jacob D. Scott, Matthew N. Jackson, Rachel E. Moffat, Monterey D. Domike, Abbey R. Larson, George Higgins, Payson C. Danielson, Hyunbi Hwang, Matthew J. East, Ethan Walbom, Shule M. Aggabao, Harry M. Peless, Elisa A. Correa Lazaro, Jayden S. Longhurst, Kyla Radke, Spencer T. Payne, Hayzen H. Chamberlain, Bartel Van Oostendorp, Christopher C. Harrell, Austin M. Johnson, Natalie A. Olsen, Parker Danielson, Thomas Wilhite, Jeffrey K. Schachterle, Staci Avery, Donald P. Breakwell, Brett E. Pickett

**Affiliations:** 1 Department of Microbiology & Molecular Biology, Brigham Young University 6756, Provo, Utah, USA; Portland State University, Portland, Oregon, USA

**Keywords:** bacteriophages

## Abstract

Thunderbird, Gwilliam, and Jiraiya are mycobacteriophages in the B1 subcluster that infect *Mycobacterium smegmatis* strain *mc²155*. The genomes of these three phages are ~69 kb in length. Studying these phages will enhance our understanding of subcluster B1 mycobacteriophages, their genes, their putative functions, and potential impact as mycobacterial therapeutic agents.

## ANNOUNCEMENT

To better understand both the potential impact of mycobacteriophages as therapeutic agents and the diversity of mycobacteriophages in local soil (1), the three phages in this study were isolated from soil samples taken in Provo, Utah (Table 1). An established protocol was used for phage isolation and purification. Briefly, 5 mL of soil was placed in a 15 mL conical vial and suspended in 8–10 mL Middlebrook 7H9 broth prior to mixing by inverting five times and filtering through a 0.45 µm filter (2). A 500 µL aliquot was used to infect 250 µL of log phase *Mycobacterium smegmatis* mc^2^155 host cells, mixed with top agar then plated on 7H10 agar prior to incubation at 37°C for two days. Plaques, which were generally small, clear, and round, were picked using a sterile micropipette tip. Following at least four rounds of plaque purification, a high titer lysate (>1E10^9^ PFU/mL) was prepared by flooding nearly confluent “web-plates” with 7H9 broth, incubating for 2 h at room temperature, decanting, and filtering through a 0.22 µm filter.

**TABLE 1:** Features of the three B1 subcluster mycobacteriophages

Phage name	Thunderbird	Gwilliam	Jiraiya
GPS coordinates	40.245316 N, 111.650324 W	40.262446 N, 111.652869 W	40.244674 N, 111.650366 W
Isolation details (year, temperature, depth)	2024, 12°C, surface	2023, 10°C, 5 cm	2023, 37°C, 3 cm
Collection material notes	Moist soil under a waterfall	A raised garden bed after a good growing season. Gathered soil after a rainstorm in the fall. Soil was still semi-dry.	Moist soil around the roots of a plant
Sequence reads (millions)	1.80	1.33	1.09
Sequence depth (× coverage)	3,382	5,474	1,814
Genome length (base pairs)	68,862	69,646	68,861
GC content (%)	66.4	66.4	66.4
Character of genome ends	Circularly permuted	Circularly permuted	Circularly permuted
Most similar phage (% ANI)	JakeO (99.01%)	Usavi (99.03%)	Thunderbird (99.83%)
# ORFs	102	102	103
# Orphams	0	0	0
# tRNAs	0	0	0
# ORFs with putative function (%)	26 (25.5)	32 (31.4)	31 (30.0)
# ORFs with no putative function (%)	76 (74.5)	70 (68.6)	72 (69.9)

DNA was extracted from the lysate by using the Norgen Phage DNA Isolation Kit following manufacturer’s protocol prior to library preparation and sequencing. Briefly, 0.5 µg of DNA per sample was used for DNA library preparation using the NEBNext Ultra DNA Library Prep Kit for Illumina (NEB, USA) following manufacturer’s recommendations, and unique index codes were added to each sample. The DNA samples were sonicated to a size of 350 bp, then DNA fragments were end-polished, A-tailed, and ligated with the full-length adaptor for Illumina sequencing with further PCR amplification. PCR products were purified (AMPure XP system), and libraries were analyzed for size distribution with an Agilent 2100 Bioanalyzer and quantified using real-time PCR.

The genomes were sequenced using Illumina NovaSeq X PE150 with the corresponding sequencing kit, generating 1.09–1.80 million 150 bp paired-end reads per genome. Reads were trimmed using TrimGalore v0.6.6 (3) with a minimum length of 20 bases and minimum phred quality score of 20. Trimmed reads underwent assembly with Newbler v2.9 (4), prior to validation and evidence of genome circularization via reads spanning the 5′ and 3′ ends of the consensus sequence using CONSED v2.9 (5) with default parameters.

**Fig 1:**
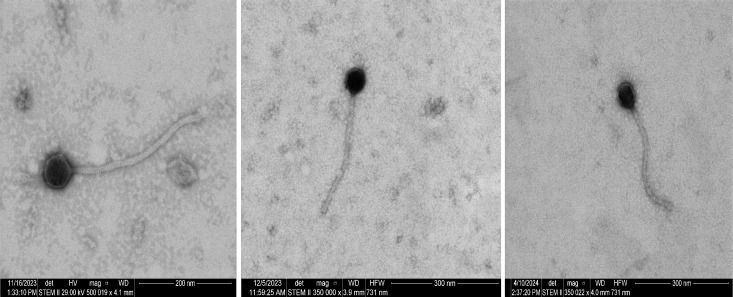
Scanning-transmission electron micrographs of the Thunderbird (left panel), Gwilliam (center panel), and Jiraiya (right panel) phages, with 20–29 kV and an 86 pA probe. Micrographs were collected using a STEM II detector and an ion beam with a brightfield light in immersion mode at ~350,000×–500,000× magnification on a Helios Nanolab 600 FEI instrument.

Fasta files of each genome sequence were first auto-annotated by the Genemark 3.0 (6) and Glimmer 2.5 (7) algorithms within DNA Master (8) using default parameters. These gene annotations were then manually verified using default parameters for Starterator, Phamerator genome maps (9), tRNAscanSE tRNA analysis (10), HHPRED (11), GeneMarkS coding potential maps (12), PhageScope (13), and BLASTp (14). Using BLAST similarity and phagesDB annotations (https://phagesdb.org/) (15), these three phages were assigned to the B1 subcluster. Negative-stained scanning transmission electron microscopy (STEM; 2% uranyl acetate) imaging showed that these phages have a siphovirus-like morphotype (Fig. 1).

BLASTN and fastANI identified the most similar phages to these three (16). Thunderbird has 102 ORFs, 26 encode proteins with predicted function (25.5%); Jiraiya has 103 ORFs, 31 with predicted function (29.8%); Gwilliam has 102 ORFs, 32 with predicted function (32.0%). We found no tRNAs, tail assembly chaperone frameshifts, or orphams in these phages. We observed one ORF, predicted to encode a 119 amino acid protein that was present in both Thunderbird (ORF 54) and in Jiraiya (ORF 55) but not Gwilliam.

## Data Availability

Thunderbird is available at GenBank with Accession PQ114757 and Sequence Read Archive (SRA) with accession SRX27013408. Gwilliam is available at GenBank with Accession PV105560 and Sequence Read Archive (SRA) with accession SRX27013411. Jiraiya is available at GenBank with Accession No. PV105564 and Sequence Read Archive (SRA) with accession SRX27013413.
